# Room for Improvement: How does the Media Portray Individuals Who Engage in Material Depicting Child Sexual Abuse?

**DOI:** 10.1007/s12119-022-09945-x

**Published:** 2022-03-05

**Authors:** Larissa S. Christensen, Katarina Pollard

**Affiliations:** 1 Sexual Violence Research and Prevention Unit, School of Law and Society, USC Australia, 90 Sippy Downs Drive, 4558 Maroochydore, DC, QLD Australia; 2 School of Law and Society, USC Australia, 90 Sippy Downs Drive, 4558 Maroochydore, DC, QLD Australia

**Keywords:** Child sexual abuse material, Child exploitation material, Child sexual abuse, Child sex offender, Media

## Abstract

The media has a powerful ability to shape public perceptions. Given the current disjuncture between social attitudes and the law in relation to child sexual abuse material (CSAM) it is important to explore the representation of CSAM offenders in the media. The aim of this exploratory study was to identify how CSAM offenders are depicted in the print media. Newspaper articles published from 1 August 2019 to 31 August 2020 on CSAM offenders in Western countries were searched. A qualitative content analysis was employed, revealing three themes across the 56 articles: (1) the headings of the articles downplayed the abusive offending; (2) offender’s motivations indicate heterogeneity in offending; and (3) sentencing remarks communicated denunciation and the harms of offending. While the first theme identified that there is still space for improvement in the reporting of these crimes, the second theme indicates the various motivations portrayed should assist the public in identifying that these offenders are heterogenous in their motivations, thus potentially countering some previously held stereotypes. Finally, the denunciation and the harms taken from the messages delivered by the judiciary in the sentencing remarks could offer a positive educational tool for some members of the public. These findings should be valuable for media, law enforcement, and psychology disciplines.

## Introduction

Not all members of the public may consider child sexual abuse material (CSAM) a serious crime (Warner 2010). Some members of the public may question whether some CSAM offenses are regarded as a ‘serious criminality in a modern and permissive society’ (Warner 2010, p. 395). In fact, research has found some disjuncture between social attitudes and the law in relation to CSAM (Hunn et al. 2020; Ost 2002; Prichard et al. 2015a; Prichard et al. 2015b). While prior research has explored the messages disseminated to the public in relation to CSAM offending, via authorized spokespersons (e.g., judiciary) who censure crime (Christensen and Tsagaris 2020; Daly and Bouhours 2008; Hunn et al. 2018; Prichard et al. 2015b), little research has explored the messages disseminated through the media on CSAM offending. As the media has the powerful ability to shape public perceptions (Cole and Daniel 2005; Gray 2013) it is therefore imperative to explore the representation of CSAM offenders in the media, which this study sets out to do. These findings should be a valuable resource for law enforcement, media, and psychology disciplines.

### Literature Review

The CSAM industry has rapidly increased throughout history with the material produced exploiting children through many means, including physical and sexual abuse (Jenkins 2001; Ost 2002; Taylor and Quayle 2003). This rapid increase is partly due to technological advances, enhancing one’s ability to produce, access, and distribute CSAM (Henshaw et al. 2018). In particular, the internet is an influential factor in the enablement of accessing and distributing CSAM as the internet is easy to *access*, *affordable*, and provides *anonymity*; referred to as the Triple A engine (Cooper, 1998). The proliferation of the CSAM industry has also seen rise during the COVID-19 pandemic due to travel and contact restrictions, and the number of clients and market will likely continue to increase in the near future (European Union Agency for Law Enforcement Cooperation 2020).

While CSAM offenders are a heterogenous offending group, they appear to hold a few key psychological and demographic characteristics. Compared with contact child sexual offenders, CSAM offenders tend to have fewer cognitive distortions, have less emotional identification with children, and higher rates of victim empathy and deviant sexual interests (Babchishin et al. 2011; Babchishin et al. 2015; Merdian et al. 2013). In terms of demographic characteristics, CSAM offenders are more likely to be the racial majority and younger than contact child sexual offenders (Babchishin et al. 2011; McCarthy 2010; Wolak et al. 2005). Compared with mixed (both contact and CSAM) and contact child sexual offenders, they are more likely to be in professional employment and are more educated (Lee et al. 2012).

In terms of CSAM victims, female children and adolescents are at a significantly higher risk of attracting CSAM offenders than their male counterparts (Bang et al. 2014). CSAM victims are often exposed to physical and sexual harm during the production which can lead to future psychological ramifications as the victims can see themselves in the way that they are represented in the abusive content (Ost 2002). In addition to the abusive event, victims of CSAM can endure ongoing pain and trauma well into the future (Leary 2007) and the permanent record of the abuse continues to victimize the child every time to material is consumed (Beech et al. 2008). Child victims of CSAM offenses have their rights to life, liberty, and security undermined which can also cause physical and psychological harm (Ost 2002).

The public is dependent on the media to receive a substantial proportion of their information. The mass media is empowering and has a significant influence on society’s understanding and opinion of CSAM offenders (Ball-Rokeach and DeFleur 1976; Christensen 2018; Jackob 2010; Tai and Sun 2007). Not only can the misconstruction of certain types of offenses through the media marginalize victims (Hetherton 1999) it can obstruct victims from reporting the offenses (Hayes and Baker 2014). Beyond victims, public perceptions are also integral to the reintegration and rehabilitation outcomes of CSAM offenders due to labelling theory which results in social, economic, and psychological consequences of the offenders (Schultz 2014). Despite some negative impacts that the media can have, there are some positive impacts. For instance, the media can be utilized as a preventative strategy to deter offenders (Smallbone and Wortley 2017); the publicization of arrests and sentences in the media can deter some offenders (Baron and Kennedy 1998).

Given the media’s powerful ability to shape public perceptions (Cole and Daniel 2005; Gray 2013) and the current disjuncture between social attitudes and the law in relation to CSAM (Hunn et al. 2020; Ost 2002; Prichard et al. 2015a; Prichard et al. 2015b), it is therefore imperative to explore the representation of CSAM offenders in the media. Further, there have been calls in the research to examine media reporting of CSAM and, in particular, the sentencing (Berry et al. 2012; Hunn et al. 2018; Roberts and Doob 1990). To the authors’ knowledge, no study to date has explored the portrayal of male CSAM offenders in the print media. The aim of this exploratory study was to identify how CSAM offenders are depicted in the print media. Such findings should be valuable across media, law enforcement, and psychology disciplines.

## Method

A keyword search was conducted by the second author using the sampling frame of 1st August 2019 to 31st August 2020 on the EBSCO server.^1^ This sampling frame allowed for the most up-to-date sample of articles. Search terms used were: “child pornography” OR “child porn” OR “child exploitation material” OR “child sexual exploitation material” OR “porn” OR “child sexual abuse material” OR “child abuse material” OR “child exploitation online” OR “kiddie porn”. Inclusion criterion were that the articles had to: discuss a specific CSAM case rather than CSAM more generally; be published in English; and be published in a Western country.^2^ The search resulted in 283 articles across the 12-month period.^3^ Many cases were removed due to the articles not containing sufficient information to determine whether the offenses were specifically related to children due to the search term “porn”, a general focus on CSAM that did not detail a specific case, or replication of the same article. The final sample was comprised of 56 articles, and was reviewed by the first author to confirm all articles met the inclusion criteria.

### Analytical approach

In line with prior studies that have explored media representations of child sexual offenders (Christensen 2018; Landor and Eisenchlas 2012), this study utilized a qualitative content analysis. The focus of this systematic approach allows researchers to “document and understand the communication of meaning” (Altheide 1987, p. 68). A qualitative content analysis was selected as the purpose of the study was to explore the communication of *meaning* in the portrayal of CSAM offenders as opposed to a quantitative content analysis focused on the *occurrence* of themes (Altheide 1987). The first author utilized Altheide’s (1987, p. 71) process, and read each newspaper article without “predefined and rigid categories” to identify what was relevant. Messages were assessed across articles, with general categories documented, before re-examining the articles to check the appropriate information had been documented. Through this continuous refinement process, themes were substantively informed (Altheide 1987). To ensure the credibility of the approach, the themes were reviewed by the second author.

## Results

### Characteristics of Articles

There were 43 cases of reported CSAM offending across 56 articles. Most cases involved one article, except five cases which involved several similar write-ups of the same case (minimum = 1 article per case, maximum = 5 articles per case, *M* = 1.30 articles). Each case involved one CSAM offender (N = 43 offenders). All offenders were male, except for two females. Of known cases, offenders ranged in age from 21 to 82 years (*M* = 43.09 years) at the time of crime commission.^4^ The youngest offender was a teenager who was still in high school, but his age was not provided. Employment status was indicated in less than half of the cases. Very few of these offenders had blue collar jobs (e.g., butcher, bus driver, manager of a restaurant) with most offenders having white collar jobs (e.g., surgeon, lecturer, former Member of Parliament, deputy principle). Only two cases indicated some mental vulnerabilities (depression and lacking emotional intelligence), while three cases referred to drug use, depression, or alcoholism.

Of known cases, the offending was typically discovered through joint operations by authorities, tip-offs to police, undercover operatives, search warrants, or through reports by members of the public. For example, a computer shop technician discovered the images on the offender’s device, while in another case a teacher located the images on the offender’s phone. In another instance, the offender informed a nurse (mandated reporter) that he was attracted to young girls. Acknowledging that not all cases provided a definitive number, the average number of images and videos offenders had was 1,670 (minimum = 1 video/image, maximum = 207,000 videos/images). The child victims appeared to range in age spanning infants through to pubescent children, and there appeared to be a slight preference towards female children.

Most offenders were charged with one offense (minimum = 1 charge, maximum = 3 charges, *M* = 1.61 charges). These offenses predominantly surrounded access / possession offenses, followed by production / making offenses, and transmitting / distributing offenses. In only seven cases the offender was charged with an additional offense, these included drug and weapon offenses, sexual abuse and sexual assault offenses, and obstructing a police officer. Of known cases, all offenders had pleaded guilty except for one offender.^5^ About half of the cases had been prosecuted to date. Most (42%) of these offenders received a suspended prison sentence (minimum = 9 months, maximum = 36 months, *M* = 20.29 months), followed by a non-suspended prison sentence (26%, minimum = 15 months, maximum = 36 months, *M* = 24 months), and other sentences (21%, e.g., community work, community-based correction order). In the remaining 11% of cases, the sentence was unknown, or the charges were cleared. Criminal history was highlighted in 14% of cases, these prior offenses included CSAM, child sexual abuse, and drug offenses.

### Themes

#### The headings of the articles downplayed the abusive offending

Despite emerging reluctance among those in policy and practice on using the term ‘child pornography’ due to the abusive nature being trivialized within this term, all articles except four referred to ‘child pornography’. In fact, many of the headings of the articles simply referred to ‘child porn’ or ‘porn’, such as ‘child porn guilty plea’, ‘facing jail over sick child porn’, ‘police bust vile pervert with more child porn’, and ‘infant in his dad’s porn vids’. A number of headings even referred to the material as ‘kid porn’ such as, ‘kid porn charge’, ‘jail boss in court over kid porn’, and ‘doctor kid porn charges’. Such terms are problematic as they fail to capture the exploitation and abuse within the horrific imagery (Leary 2007). This could, in turn, impact how the public perceive the offending. It could also further trivialize and rationalize the offending by those offenders who already discount the harm of their offending. Only four articles appeared to refer to the ‘abuse’ or similar in the headings, using terminology that did not trivialize the abuse: ‘Ex-firey faces child abuse material charges’, ‘top policewoman had video of child abuse on her phone’, ‘man who had child sex videos and pictures avoids prison’, and ‘woman avoids prison over child abuse video’.

#### Offender’s motivations indicate heterogeneity in offending

Many cases referred to the offender’s motivations for their offending. These cases appeared to concern: (1) downplay or disassociation, (2) curiosity, and (3) sexual attraction. Across most of these cases, despite the high number of guilty pleas, offenders appeared to mostly downplay or disassociate from their offending. For example, in one case, the offender intentionally sent money overseas on various occasions for CSAM, yet separated himself from the offending suggesting he would drink often and did not know what he did when intoxicated. Another offender rationalized his offending during the police interview, suggesting most men think about rape fantasy. A different offender suggested he viewed the material to relive his own childhood abuse. In one case a school deputy principal indicated he fantasized about the abuse as opposed to being ‘into it’ and that he was not interested in the abusive material because of his work and that it revolted him.

In other cases, the offenders suggested they were curious about the material. For example, in one case the offender suggested he originally accessed the material by accident when finding adult pornography and that curiosity led him to look at it. In another instance, the elderly offender admitted to finding the material ‘interesting’. While in a different case the offender suggested he searched for the material out of ‘boredom’. Finally, in other cases, the offenders were forthright and acknowledged their sexual attraction or admitted they were ‘excited’ and aroused by the material. For example, one offender admitted to the activity being illegal and that he accessed it for sexual gratification. In another instance the offender first lied out of panic, suggesting he accessed the material due to research in his job as a social worker, before being forthright in his admissions since that point. In these instances, the offenders had typically taken active steps by commencing treatment with an expert, or indicating they felt guilt and shame and wanted to understand their offending, so they did not engage with the material again.

#### Sentencing remarks communicated denunciation and the harms of offending

Many of the articles noted some of the sentencing remarks spoken by the Judge or Magistrate during the sentencing hearing. The judiciary made reference to the need for denunciation: the public condemnation for the unacceptable offending behavior. For example, one Judge referred to the offending as “vile conduct” and that it was feeding a “deplorable demand” while another referred to it as “disturbing, gross and vile”. It was evident the judiciary also made a concerted effort to communicate to the offender, victim, and society about the harms of CSAM. One Judge stated, “children have been harmed to satisfy the perverse desires of people like you” while describing the offending as “repugnant”. Another indicated that the punishment needed to denounce the offending given the market and demand of CSAM. Overall, the judiciary indicated that the material is not a victimless crime and that acts of accessing such material leads to new material being produced. As one Judge stated, such abuse happens “because there are people like you who want to watch these films”.

## Discussion

The current study set out to identify the portrayal of CSAM offenders in the print media. It is imperative to explore the representation of CSAM offenders as the media has the powerful ability to shape public perceptions (Cole and Daniel 2005). Three themes were identified: (1) the headings of the articles downplayed the abusive offending; (2) offender’s motivations indicate heterogeneity in offending; and (3) sentencing remarks communicated denunciation and the harms of offending. These themes suggest that while developments can be made in the language used in the headings, the public can at least understand that this offending group is heterogenous in their motivations (potentially countering any previously held stereotypes) and are exposed to the denunciation delivered by the judiciary. The findings from the current study are discussed below, along with implications, limitations, and ideas for future research.

### Characteristics

Age of offenders (*M* = 43.09 years) was consistent with previous research. For example, Christensen and Tsagaris (2020) found CSAM offenders were 40.61 years on average at time of sentencing. Unlike previous research that found diversity across occupation (e.g., Christensen and Tsagaris 2020; Tsagaris et al. 2017), the current study found most offenders worked in white collar jobs; many of the offenders were in a position of power and trust for example law enforcement, education, and the medical field. While the current findings might indicate the changing demographics of these offenders, another reason for this finding might be that these cases were reported as they were perceived to be more newsworthy. For example, a surgeon being charged with CSAM could be considered to be more newsworthy than an individual who is unemployed. However, Christensen and Tsagaris (2020) did find a subgroup of offenders in their study that appeared to live a double life; these individuals were well-esteemed within their public life, which greatly differed to their private life. Regardless, the current finding is positive as it may possibly target any pre-existing stereotypes of CSAM offenders held by the public, by highlighting well-esteemed individuals can live unsuspecting parallel lives. It may also send the message to any well-esteemed individuals considering this offending that prior good character is not a rarity for this offending and is therefore of less significance during sentencing compared with other crimes (Christensen and Tsagaris 2020). One idea for future research could involve exploring clinical samples of CSAM offenders and identifying whether the motivations to engage in offending differ across employment type: white collar, blue collar, and unemployed.

The finding that most cases predominantly involved possession offenses was in line with prior research (Christensen and Tsagaris 2020; Jung and Stein 2012). The high guilty plea in the current study was also consistent with previous research. For example, Jung and Stein (2012) found 92% of offenders pleaded guilty and Christensen and Tsagaris (2020) found 93% of cases involved a guilty plea. Also similar with previous research was the lack of cases that reported criminal history (Babchishin et al. 2011; Christensen and Tsagaris 2020; McCarthy 2010). While most offenders in the current study who had been prosecuted were handed a custodial sentence, these predominantly involved a suspended prison sentence. This finding was inconsistent with prior research (Christensen and Tsagaris 2020; Jung and Stein 2012). One reason for the current finding might be related to framing effects theory. This theory refers to the media’s ability to influence public attitudes through the way they report on an issue (Lecheler and De Vreese 2019). The frame of this reporting – reporting cases where most did not result in incarceration – is consistent with the media reporting of courts being soft on crime (Prenzler 2020).

### Themes

The first theme, *the headings of the articles downplayed the abusive offending*, uncovered a negative finding. Most headings of these articles used the term ‘child porn’ or ‘kid porn’. Scholars and advocates have made repeated attempts to highlight that the term ‘child pornography’ undermines the gravity of the offending and trivializes the abuse (Save the Children 2005). The term fails to capture the exploitation and sexual abuse of a child that is depicted in the dreadful imagery (Leary 2007). While it is acknowledged that headings are restricted by word counts, ‘child abuse’ could be considered as an appropriate, and more suitable, option. Research suggests that the misconstruction of certain types of offenses through the media can marginalize victims (Hetherton 1999) and obstruct victims from reporting the offenses (Hayes and Baker 2014). Therefore, reframing the material as abusive may assist these victims in disclosing and reporting the abuse. Such reframing is also particularly important when there appears to be a disjuncture between social attitudes and the law in relation to CSAM (Prichard et al. 2015b). Research has found some members of the public do not perceive any further harm is perpetrated from viewing CSAM beyond the production of the material (overseeing the increased demand and the ongoing trauma and distress for victims) (Prichard et al. 2015b). Therefore, reframing the material as ‘abusive’ could assists in both challenging perceptions among the public and assisting victims of CSAM offending in coming forward to report the abuse.

The second theme, *offender’s motivations indicate heterogeneity in offending*, revealed some suggested drives behind the offending. Three distinct motivations arose across the cases: (1) downplay or disassociation, (2) curiosity, and (3) sexual attraction. *Downplay or disassociation* appeared to be the most prevalent, with offenders attempting to separate themselves from their offending and engaging in rationalizations for using the material. The act of separation, or self-distancing, has previously been noted through interviews with CSAM offenders in which offenders attempts to minimize accountability and downplay their offenses (Winder and Gough 2010). While the current finding was particularly interesting given the high number of guilty pleas, it was consistent with Christensen and Tsagaris (2020) who found most CSAM offenders downplayed their offenses despite the high rate of guilty pleas.

It was not surprising to find *curiosity* as another motivation for offending particularly when CSAM offenders have been found to claim curiosity in both police samples (40%) and clinical samples (27%) for accessing the material (Seto et al. 2010). Finally, the motivation of *sexual attraction* to engage in CSAM was a predictable finding. It was interesting to note that these offenders had taken active steps to work through their offending compared with the other groups. While the authors’ are not suggesting that these are the three definitive motivations for CSAM offending (due to the limitations of the sample), this reporting challenges misconceptions of why someone might engage in CSAM. In particular, the reporting of these articles may combat any stereotypes held by the public – that only individuals with ongoing pedophilic tendencies engage in this type of offending – with the public noting the heterogeneity in the motivations behind offending. This heterogeneity also reaffirms the need for tailored treatment, particularly when risk and criminogenic needs can be diverse across CSAM offenders (Wortley and Smallbone 2012).

The final theme, *sentencing remarks communicated denunciation and the harms of offending*, was a positive finding. This is because sentencing remarks have the ability to communicate messages on the harms of offending to the offender, victim, and society. This also includes the judiciary elucidating why certain behavior is punishable through criminal sanctions (Hunn et al. 2018). In turn, Hunn et al. (2018) argue that sentencing remarks can be a valuable educational tool and a form of crime prevention, as the sentencing remarks offer a primary source of messaging on the wrongs of viewing CSAM, which can have public outreach such as through media coverage. Prior research has found the reporting of Judges’ remarks from sentencing hearings as a useful tool for conveying messages in media articles (Christensen 2018). Such public messaging is imperative when there appears to be disjuncture between social attitudes and the law in relation to CSAM (Prichard et al. 2015b). Therefore, given that a number of articles communicated the denunciation and the harms delivered by Judges, these messages could act as a positive educational tool for some members of the public.

### Limitations

This study has several limitations. First, while the characteristics of CSAM offenders were detailed and offer some interesting findings, these characteristics are not generalizable to this group of offenders. This is because the sample involved newspaper articles, and certain cases may have been selected for reporting over other cases due to elements of newsworthiness. However, the purpose of the study was not to provide a representative depiction of the characteristics per se, but rather, to offer insight into the characteristics of those *reported* cases. In doing so, it revealed the offending group as heterogenous, which is important for the public to recognize, challenging any misconceptions that CSAM offenders have a particular profile. Second, the study did not explore how the portrayal of CSAM offenders has changed over time. While this would have offered some interesting insights, this was not the purpose of the exploratory study.

## Conclusions

The aim of this exploratory study was to identify how CSAM offenders are depicted in the print media. A number of interesting characteristics were identified. For example, most offenders had white collar jobs and received suspended prison sentences. While the authors’ acknowledge this sample is not representative of all CSAM offenders, it challenges misconceptions that might be held by some members of the public. The qualitative content analysis identified three themes: (1) the headings of the articles downplayed the abusive offending; (2) offender’s motivations indicate heterogeneity in offending; and (3) sentencing remarks communicated denunciation and the harms of offending. The first theme suggests there is still space for improvement in the reporting of these crimes. Through reframing terms such as ‘child porn’ to ‘child abuse’ could enhance public perceptions on the severity of CSAM offending as well as assist victims of CSAM to disclose and report the offending. While developments are required in the language used in the headings, the portrayal of these offenders should assist the public in identifying that this offending group is heterogenous in their motivations (potentially countering any previously held stereotypes) and the denunciation and harms outlined by Judges can offer a positive educational tool for some members of the public.

## Data Availability

Not applicable.

## Footnotes

1 One keyword search was conducted across eighteen EBSCO databases including MasterFILE Premier. The ‘publication type’ was set to ‘newspaper’ to ensure other publication types did not appear (e.g., books, journal articles, conference proceedings etc.).

2 The focus was on Western countries given most research to date on CSAM offenders has been conducted in Western countries.

3 While 283 articles appeared on the EBSCO server, it is acknowledged that more articles could have appeared had additional search engines been utilized.

4 If offenders perpetrated offenses over several years, the age when the offending first started was used.

5 In this case the offender pleaded guilty to some offenses and not guilty to other offenses.
